# Simultaneous inhibition of the estrogen receptor and 6-phosphofructo-2-kinase (PFKFB3) for the treatment of ER+ breast cancer

**DOI:** 10.1186/2049-3002-2-S1-P29

**Published:** 2014-05-28

**Authors:** Yoannis Imbert-Fernandez, Sucheta Telang, Jason Chesney

**Affiliations:** 1 JG Brown Cancer Center, University of Louisville, Louisville, KY, USA

## Background

The family of 6-phospofructo-2-kinase/fructose-2,6-bis-phosphatases (PFKFB1-4) control glycolytic flux via their product, fructose-2,6-bisphosphate (F26BP), which activates the key control step, 6-phosphofructo-1-kinase (PFK-1). We recently demonstrated that exposure of estrogen receptor (ER)+ MCF-7 human breast cancer cells to estradiol (E2) causes a rapid increase in ^14^C-glucose uptake and glycolysis that is coincident with an induction of PFKFB3 mRNA (via ER binding to its promoter), protein expression and the intracellular concentration of its product, F26BP. Selective inhibition of PFKFB3 expression and activity using siRNA or a PFKFB3 inhibitor, PFK158, markedly reduced the E2-mediated increase in F26BP, ^14^C-glucose uptake and glycolysis. Importantly, co-treatment of MCF-7 cells with PFK158 and an ER antagonist synergistically induced apoptotic cell death *in vitro*. In the current study, we sought to determine if co-administration of PFK158, with ICI 182,780 (fulvestrant), would have an additive anti-tumor effect on ER+ human breast cancer xenografts *in vivo*.

## Materials and methods

Methods included apoptosis assays, measurements of ^14^C-glucose uptake and glycolysis, and an E2-dependent ER+ MCF-7 xenograft model of breast tumorigenesis.

## Results

Initially, we found that the inhibitory effects of PFK158 on MCF-7 cell glycolysis but not glucose uptake lasted 24 hours after E2 exposure suggesting that flux through PFK-1 and not the pentose shunt was the key metabolic target of PFK158. We then confirmed that E2 upregulated PFKFB3 in a second ER+ breast cancer cell line, T47D cells, and found that PFK158 also synergistically increased the apoptotic effects of ICI 182,780 in these cells. In order to determine if there exists potential clinical utility in combining PFKFB3 and ER antagonists, we next implanted BALB/c athymic mice with 17beta-E2 pellets (dose 1.7 mg/60 days) and subcutaneously injected 10^7^ MCF-7 cells in 100 μl of matrigel. After 7 days, mice that developed tumors >80 mg were randomized into four treatment groups and administered DMSO, ICI 182,780, PFK158 or the combination and tumor volumes were assessed every three days for 45 days using microcalipers. We found that the combination of ICI 182,780 with PFK158 yielded the greatest anti-tumor effect in this E2-dependent model of tumorigenesis: DMSO, 298 ± 62.86 mg; ICI 182,780, 160 ± 62.4 mg; PFK158, 142 ± 90 mg; ICI 182,780 + PFK158, 45 ± 38 mg.

## Conclusions

These results provide essential pre-clinical information that may allow for the design of combinatorial phase I/II trials of PFKFB3 antagonists with anti-estrogen therapies in ER+ stage IV breast cancer patients.

